# Stigma towards borderline personality disorder: effectiveness and generalizability of an anti-stigma program for healthcare providers using a pre-post randomized design

**DOI:** 10.1186/s40479-015-0030-0

**Published:** 2015-05-05

**Authors:** Stephanie Knaak, Andrew CH Szeto, Kathryn Fitch, Geeta Modgill, Scott Patten

**Affiliations:** Opening Minds Anti-Stigma Initiative, Mental Health Commission of Canada, 110 Quarry Park Blvd, Suite 320, T2C 3G3 Calgary, AB Canada; Department of Psychology, University of Calgary, 2500 University Dr. NW, T2N 1 N4 Calgary, Alberta Canada; Department of Psychiatry, University of Calgary, DBT Program & Central Mental Health Clinic and Foothills Medical Centre, Calgary Zone, Alberta Health Services, Calgary, Canada; Department of Community Health Sciences, University of Calgary, 4th Floor TRW Building, 3280 Hospital Drive NW, T2N 4Z6 Calgary, Alberta Canada; Department of Psychiatry & Mathison Centre for Mental Health Research & Education, University of Calgary, 4th Floor TRW Building, 3280 Hospital Drive NW, T2N 4Z6 Calgary, Alberta Canada

**Keywords:** Stigma, Borderline personality disorder, Healthcare providers, Anti-stigma program, Psycho-education intervention, Dialectical behavior therapy, Social contact

## Abstract

**Background:**

Stigmatization among healthcare providers towards mental illnesses can present obstacles to effective caregiving. This may be especially the case for borderline personality disorder (BPD). Our study measured the impact of a three hour workshop on BPD and dialectical behavior therapy (DBT) on attitudes and behavioral intentions of healthcare providers towards persons with BPD as well as mental illness more generally. The intervention involved educational and social contact elements, all focused on BPD.

**Methods:**

The study employed a pre-post design. We adopted the approach of measuring stigmatization towards persons with BPD in one half of the attendees and stigmatization towards persons with a mental illness in the other half. The stigma-assessment tool was the Opening Minds Scale for Healthcare Providers (OMS-HC). Two versions of the scale were employed – the original version and a ‘BPD-specific’ version. A 2x2 mixed model factorial analysis of variance (ANOVA) was conducted on the dependent variable, stigma score. The between-subject factor was survey type. The within-subject factor was time.

**Results:**

The mixed-model ANOVA produced a significant between-subject main effect for survey type, with stigma towards persons with BPD being greater than that towards persons with a mental illness more generally. A significant within-subject main effect for time was also observed, with participants showing significant improvement in stigma scores at Time 2. The main effects were subsumed by a significant interaction between time and survey type. Bonferroni post hoc tests indicated significant improvement in attitudes towards BPD and mental illness more generally, although there was a greater improvement in attitudes towards BPD.

**Conclusions:**

Although effectiveness cannot be conclusively demonstrated with the current research design, results are encouraging that the intervention was successful at improving healthcare provider attitudes and behavioral intentions towards persons with BPD. The results further suggest that anti stigma interventions effective at combating stigma against a specific disorder may also have positive generalizable effects towards a broader set of mental illnesses, albeit to a lessened degree.

## Background

Stigmatization among healthcare providers towards persons with a mental illness is believed to present obstacles to effective caregiving [1-4]. This may be especially the case for persons with borderline personality disorder (BPD), where it has been suggested that negative reactions can lead to counter-therapeutic conditions including premature termination of treatment, rationalization of treatment failures, a lower likelihood of forming an effective treatment alliance with patients, emotional and social distancing, difficulty empathizing, a lack of belief in recovery, and perceptions of patients as powerful, unrelenting, dangerous, manipulative and more in control of their behaviors than other patients [1,5-9].

Research has found that clinicians commonly report experiencing anger, frustration, inadequacy, and feelings of being challenged in response to patients with BPD [10,11]. Also, when comparing BPD to other highly stigmatized disorders such as schizophrenia and affective disorder for example, attitudes and behaviors towards BPD have tended to be more negative [5,6,8]. It has thus been argued that there is considerable need for education and training aimed at improving healthcare providers’ attitudes, as well as their ability to interact effectively with patients with BPD [12-14].

There is some research on psycho-education as an effective intervention for BPD with patients and family members [15-19]. There is also some research suggesting psycho-education can improve clinician attitudes [12-14]. Our study sought to contribute to this literature by examining the extent to which a three hour workshop on BPD and dialectical behavior therapy (DBT) was effective at improving attitudes and behavioral intentions of healthcare providers towards patients with BPD. Our study design also offered the opportunity to examine spillover effects of the intervention; namely, the impact of this disorder-specific intervention on attitudes towards persons with a mental illness more generally.

The program was developed by Dr. Kathy Fitch, MD, FRCPC, a specialist in DBT and a general adult inpatient psychiatrist in Calgary, Canada. The program was delivered at a Calgary hospital on November 21, 2012 by Dr. Fitch and three co-presenters to a group of 230 healthcare providers, as part of an education series targeting front-line community and outreach service providers as well as hospital-based providers. Workshop attendees were eligible to receive continuing medical education credits for their participation. Participants registered for the workshop ahead of time. Table 1 provides demographic information about program attendees.

**Table 1:** **Participant demographic information: all participants and by scale type**

	**Original OMS-HC**	**BPD-specific OMS-HC**	**All participants**
	(n = 94)	(n = 93)	(n = 187)
Gender			
Male	15 (16.0%)	13 (14.0%)	28 (15.0%)
Female	79 (84.0%)	80 (86.0%)	159 (85.0%)
Age (mean)	39.0 yrs	39.2 yrs	39.1 yrs
Occupation			
Social worker	33 (35.1%)	40 (43.0%)	73 (39.0%)
Nurse	13 (13.8%)	14 (15.1%)	27 (14.4%)
Counselor	14 (14.9%)	8 (8.6%)	22 (11.8%)
Occupational therapist	8 (8.5%)	11 (11.8%)	19 (10.2%)
Psychologist/psychiatrist	11 (11.7%)	4 (4.4%)	15 (8.0%)
Student	6 (6.4%)	3 (3.2%)	9 (4.8%)
Director/manager	5 (5.3%)	3 (3.3%)	8 (4.2%)
Other	3 (3.2%)	8 (8.6%)	11 (5.9%)
Years in practice (mean)	12.7 yrs	11.7 yrs	12.2 yrs
Ever been treated for a mental illness?			
No	64 (68.1%)	67 (72.0%)	131 (70.1%)
Yes	30 (31.9%)	26 (28.0%)	56 (29.9%)
Ever been treated for BPD?			
No	90 (95.7%)	92 (98.9%)	182 (97.3)%
Yes	4 (4.3%)	1 (1.1%)	5 (2.7%)
Ever treated a person with a mental illness?			
No	13 (14.0%)	20 (21.5%)	33 (17.7%)
Yes	80 (86.0%)	73 (78.5%)	153 (82.3%)
Specialize in working with patients with BPD?			
No	80 (85.1%)	79 (85.9%)	159 (85.5%)
Yes	14 (14.9%)	13 (14.1%)	27 (14.5%)

The program’s objective was to improve healthcare providers’ attitudes and behavioral intentions towards persons with BPD through a combination of education and skills training, as well as social contact. The workshop contained a number of ingredients shown to be effective for improving attitudes among healthcare providers towards persons with mental illnesses [20], including an educational/skills training component designed to improve healthcare providers’ abilities to effectively interact with and help patients, education to correct common misperceptions, social contact in the form of a live personal testimony from a person with lived experience of BPD, an emphasis on and demonstration of recovery (including a case study which exemplified recovery and achievement), and an enthusiastic facilitator who set the tone and modeled person-first (as opposed to disorder-first) language and behavior.

Our interest in understanding the impact of this intervention on attitudes and behavioral intentions towards both persons with BPD *and* persons with a mental illness arose from larger questions about the possible generalizability [21,22] or ‘spillover effects’ of disorder-specific interventions. We were interested in informing the question about whether a generalist or specialist approach is the better strategy for anti-stigma programming [23,24], especially for disorders characterized by high levels of stigmatization. For example, if a targeted (i.e., diagnosis-specific) intervention is effective at reducing stigma towards the specific disorder and its generalizability to other disorders and/or to mental illness more generally is neutral or positive, the relevance and value of developing and delivering such programming may be increased. However, if spillover effects are negative, a determination of program success becomes decidedly more complex.

## Methods

Our interest was to examine the extent to which the intervention led to a change in perceptions towards persons with BPD, as well as towards persons with a mental illness more generally. The study employed a pre-post design. We adopted the approach of measuring stigmatization towards persons with BPD in one half of the attendees and stigmatization towards persons with a mental illness in general in the other half. The stigma-assessment tool used was the Opening Minds Scale for Healthcare Providers (OMS-HC) [25,26], a validated scale that measures health care providers’ attitudes and behavioral intentions towards mental illness and persons with a mental illness. To complete the scale, respondents are asked the extent to which they agree or disagree with a series of items on a 5-point scale: *strongly agree (1), agree (2), neither agree nor disagree (3), disagree (4),* or *strongly disagree (5)*. To create a total scale score for the OMS-HC, all 15 items are summed for each participant with appropriate items reverse scored. Total scores can range from 15 to 75, with lower scores indicating less stigma.

The scale also captures three main dimensions of stigma: negative attitudes, willingness to disclose/seek help, and preference for social distance [26]. An example of an item from the ‘negative attitudes’ subscale is the statement, “I am more comfortable helping a person with has a physical illness than I am helping a person who has a mental illness.” An example of an item from the ‘willingness to disclose’ subscale is the statement, “If I had a mental illness, I would tell my friends.” An example from the ‘preference for social distance subscale’ is the statement, “If a colleague with whom I worked told me they had a managed mental illness, I would be just as willing to work with him/her.”

Because the intention was to measure the program’s impact on stigmatizing attitudes against persons with a mental illness as well as BPD, two versions of the scale were employed and randomly given to participants – the original version and a ‘BPD-specific’ version. The ‘BPD-specific’ version replaced each instance of the phrase ‘mental illness’ (which occurs in each item on the scale) with the term ‘borderline personality disorder’. For example, the statement, ‘If a colleague with whom I work told me they had a mental illness, I would be just as willing to work with him/her’ was changed to, ‘If a colleague with whom I work told me they had borderline personality disorder, I would be just as willing to work with him/her.’ Demographic information, including gender, age, occupation, average years in practice, and previous experience with mental illness and BPD was also collected. Ethics approval was granted by the Conjoint Health Research Ethics Board at the University of Calgary.

An assumption invoked with the study design is that the modified scale measured stigma against BPD whereas the original version of the scale measured mental illness in general. Cronbach’s alphas for both versions of the scale were calculated. Alphas for the original OMS-HC scale were .73 at pre-test and .81 at post-test. For the BPD-specific version alphas were .79 at pre-test and .80 at post-test, indicating an acceptable level of internal consistency for both versions of the scale at both time points.

The main outcome measures were the total overall and subscale OMS-HC scores at pre and post time points. Respondents with more than three missing items were dropped from the analysis. Missing items were filled with each respondent’s mean item value. Examination of a histogram and QQ plot confirmed that scores were distributed normally. To assess the overall effectiveness of the presentation on stigma towards persons with a mental illness and towards persons with BPD, a 2×2 mixed model factorial analysis of variance (ANOVA) was conducted on the dependent variable, stigma score. The between-subject factor was survey type (OMS-HC versus ‘BPD-specific’ OMS-HC) and the within-subject factor was time (pre intervention versus post intervention). Data were analyzed using PASW 18 [27].

## Results

Of the 230 participants registered for the session, 191 pre and post surveys were completed (94 paired OMS-HC surveys; 97 paired ‘BPD-specific’ surveys), representing a response rate of 83%. Participant demographics by survey type showed a high degree of similarity between the two sub-samples, suggesting the randomization process worked as designed (Table 1).

The mixed-model ANOVA produced a significant between-subject main effect for survey type (comparing stigma scores for persons with borderline personality disorder against stigma scores towards persons with a mental illness), *F* (1,189) = 39.63, *p* < .001, ƞ^2^_partial_ = .173, with stigma towards persons with borderline personality disorder (M = 35.20, SE = .61) being greater than that towards persons with a mental illness (M = 29.81, SE = .60). The ANOVA also produced a significant within-subject main effect for time, *F* (1,189) = 72.46, *p* < .001, ƞ^2^_partial_ = .277, with participants showing significant improvement in stigma scores at Time 2 (post intervention M = 31.05, SE = .46; pre intervention M = 33.96, SE = .47). The main effects were, however, subsumed by a significant interaction between time and survey type, *F* (1,189) = 28.71, *p* < .001 ƞ^2^_partial_ = .132.

**Figure 1:**
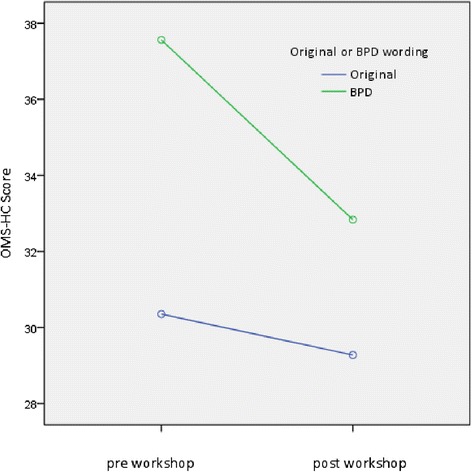
**Pre-post OMS-HC score: original scale and BPD version.**

Bonferroni post hoc tests indicated participants had greater reduction in stigmatizing attitudes towards persons with borderline personality disorder than they did towards persons with a mental illness more generally, although stigma towards both mental illness and BPD were significantly reduced (pre intervention BPD score, M = 37.56, SE = .66; post intervention BPD score, M = 32.83, SE = .65, *p* < .001; pre intervention mental illness score, M = 30.35, SE = .65; post intervention mental illness score, M = 29.28, SE = .64, *p* = .026). These results are illustrated in Figure 1.

Results for the three subscales replicated the results of the full scale (Table 2). Similarly, post hoc tests showed greater improvements towards persons with BPD than towards persons with a mental illness more generally on all three subscales (Table 3). While significant improvements were observed on all three factors for the BPD-version of the scale, only preference for social distance showed a significant improvement from pre to post workshop on the original scale (Table 3).

**Table 2:** **Mixed model ANOVA results for OMS-HC subscales: time and survey type**

	**Time**		**Survey type**		Time* survey interaction	
	**Mean (SE)**	**Assessment of significance**	**Mean (SE)**	**Assessment of significance**	**Mean difference (SE)**	**Assessment of significance**
Attitudes towards mental illness	T1 = 10.53 (.20) T2 = 9.51 (.19)	*F *(1,189) = 39.83 ƞ^2^_partial_ = .174 *p* < .001	Original = 8.91 (.25) BPD = 11.14 (.25)	*F *(1,189) = 40.32 ƞ^2^_partial_ = .176 *p* < .001	T1 Orig. vs. BPD =2.98 (.40) T2 Orig. vs. BPD =1.48 (.37)	*F *(1,189) = 21.96 ƞ^2^_partial_ = .104 *p* < .001
Disclosure/help-seeking	T1 = 10.84 (.19) T2 = 10.15 (.19)	*F *(1,188) = 19.47 ƞ^2^_partial_ = .094 *p* < .001	Original = 9.77 (.24) BPD = 11.22 (.25)	*F *(1,189) = 17.84 ƞ^2^_partial_ = .087 *p* < .001	T1 Orig. vs. BPD =1.94 (.38) T2 Orig. vs. BPD =0.97 (.38)	*F *(1,188) = 9.83 ƞ^2^_partial_ = .050 *p* = .002
Preference for social distance	T1 = 11.26 (.17) T2 = 10.38 (.15)	*F *(1,189) = 34.32 ƞ^2^_partial_ = .154 *p* < .001	Original = 9.35 (.20) BPD = 12.29 (.20)	*F* (1,189) = 112.53 ƞ^2^_partial_ = .373 *p* < .001	T1 Orig. vs. BPD =3.26 (.33) T2 Orig. vs. BPD =2.63 (.30)	*F *(1,189) = 4.44 ƞ^2^_partial_ = .023 *p* = .036

**Table 3:** **Pre-post mean score change for OMS-HC subscales: by survey type**

		**T1 M (SE)**	**T2 M (SE)**	***p*** **(Bonferroni)**
Attitudes towards mental illness	Original OMS-HC BPD OMS-HC	9.04 (.28) 12.02 (.29)	8.78 (.26) 10.25 (.27)	.248 < .001
Disclosure/help-seeking	Original OMS-HC BPD OMS-HC	9.87 (.27) 11.81 (.27)	9.67 (.27) 10.64 (.27)	.365 < .001
Preference for social distance	Original OMS-HC BPD OMS-HC	9.63 (.23) 12.89 (.24)	9.07 (.21) 11.70 (.21)	.008 < .001

In an exploratory analysis we examined gender, previous experience with a mental illness, and specialty in treating patients with BPD as potential moderators of the intervention’s effects. No significant interactions were found, although lower baseline scores were observed among women (both versions of the scale), healthcare providers who had previously been diagnosed with a mental illness (original OMS-HC only), and healthcare providers who specialized in treating patients with BPD (both versions of the scale).

## Discussion

Results showed stigma towards persons with BPD was significantly higher than that against persons with a mental illness more generally. Scores on the BPD-specific scale remained high, higher even than the baseline scores for mental illness more generally, even after the intervention. This is consistent with previous research on the stigma associated with persons with BPD. Our results provide additional evidence that BPD is a highly stigmatized disorder amongst healthcare providers, and lends support to the argument that there is considerable need for anti-stigma programming targeting this disorder. These findings also add further support to literature showing that stigma differs by diagnostic group [28,29]. In keeping with this, it is worth noting that the pre and post ratings seen with the modified scale were higher than any ratings previously observed in evaluations using the original form of the OMS-HC scale, while the ratings observed in this study using the original version of the scale were comparable to those observed in other evaluations [30,31].

Results suggest that the targeted intervention was successful at improving healthcare provider attitudes towards persons with BPD and a mental illness more generally, although the improvement in attitudes towards persons with a mental illness was considerably smaller than that towards persons with BPD. There are two likely reasons why the intervention proved to improve attitudes towards BPD to a greater degree than towards mental illness more generally. The first is that that the intervention was specifically designed to address BPD. Secondly, the higher stigma scores observed for BPD may be said to indicate greater severity of stigma compared to that of mental illness more generally. As such, there was more room for participant’s scores to improve after the intervention on the BPD measure.

Such results further suggest that attitudes towards a highly stigmatized disorder like BPD can be improved through relatively short interventions, if those interventions are designed and delivered properly [20]. It is also noteworthy that significant reductions in stigma were achieved with only a three hour program, even when the target audience was a group of practicing providers with significant experience of mental illness outside of this workshop. These findings are encouraging from the perspective of stigma reduction, as well as the value of psycho-education more generally as an effective intervention for BPD.

The results further suggest that anti stigma interventions effective at combating stigma against a specific disorder may also have positive generalizable effects towards a broader set of mental illnesses, albeit to a lessened degree. However, it is possible that at least some of the improvement in the group completing the standard scale was due to non-specific effects that would have occurred in the absence of an anti-stigma intervention. A controlled study would be required to assess this possibility. Although more research is required, these are encouraging findings for healthcare organizations looking for ways to combat stigma in the most effective and efficient manner possible.

These results as well as others [24,32] suggest that the generalizability of anti-stigma interventions should be a focus of future research. One may speculate, for example, that contact-based interventions that include first person narratives from people with several different disorders may be more generalizable than those that focus on just one. Also, these results raise the possibility that the impact of contact-based education in a particular setting may be greater if information and first-person narratives included in the programs relate to the disorders encountered most often in those settings. Existing evidence suggests that different diagnostic labels are likely to trigger specific sets of beliefs which, in turn can produce distinct emotional and behavioral reactions toward those being labeled [33,34]. These distinctions may be particularly important for anti-stigma programs that target health professionals, as diagnostic categories may play a larger role in structuring the attitudes and behaviors of health professionals than is the case among members of the general population. Given this, disorder-specific anti-stigma programs delivered to health care professionals may be more effective at addressing these stigma than a disorder-generic program.

There are limitations to this study. Our study did not include a control group to measure for non-specific effects on the scales. Our study’s objective was not so much to determine if the intervention had greater effectiveness than non-specific effects, but more to determine differences between two randomized sets of questionnaire responses measuring two different types of stigma. While future research with a randomized control group is important to determine more conclusively the effectiveness of the intervention, the randomization process undertaken in the current study would have protected against certain effects as social desirability bias. It should also be noted that previous research on the OMS-HC [25] did not find scores correlating strongly with social desirability.

Secondly, although alpha scores for the modified scale suggested an acceptable level of internal consistency and were similar to those observed for the original scale, the full psychometric performance of the modified scale remains under-examined. The assumption that the modified scale measured BPD is supported by face validity only. Further research on the adaptability of the OMS-HC to measure stigma against persons with particular mental disorders is a fruitful area for future research. Stigma is a complex concept with multiple facets and existing at multiple levels, which cannot be fully captured in a single measure [35,36]. Our study assessed healthcare providers’ attitudes and behavioral intentions towards persons with BPD and a mental illness more generally. It is not known how these findings translate into actual behaviors or specific interactions between healthcare providers and their clients with BPD or other mental illnesses.

## Conclusion

Although effectiveness cannot be conclusively demonstrated with the current research design, results are encouraging that the intervention was successful at improving healthcare provider attitudes and behavioral intentions towards persons with BPD. The results further suggest that anti stigma interventions effective at combating stigma against a specific disorder may also have positive generalizable effects towards a broader set of mental illnesses, albeit to a lessened degree.

## Abbreviations

BPD: Borderline personality disorder
DBT: Dialectical behavioural therapy
OMS-HC: Opening minds scale for healthcare providers

## References

[1] Aviram RB, Brodsky BS, Stanley B (2006). Borderline personality disorder, stigma, and treatment implications. Harv Rev Psychiatry.

[2] Lauber C, Nordt C, Braunschweig C, Rössler W (2006). Do mental health professionals stigmatize their patients?. Acta Psychiatr Scand.

[3] Schulze B (2007). Stigma and mental health professionals: A review of the evidence on an intricate relationship. Int Rev Psychiatry.

[4] Thornicroft G, Rose D, Kassam A (2007). Discrimination in health care against people with mental illness. Int Rev Psychiatry.

[5] Fraser K, Gallop R (1993). Nurses’ confirming/disconfirming responses to patients diagnosed with borderline personality disorder. Arch Psychiatr Nurs.

[6] Markham D, Trower P (2003). The effects of the psychiatric label “borderline personality disorder” on nursing staff’s perceptions and causal attributions for challenging behaviours. Br J Clin Psychol.

[7] Markham D (2003). Attitudes towards patients with a diagnosis of “borderline personality disorder” : Social rejection and dangerousness. J Ment Heal.

[8] Forsyth A (2007). The effects of diagnosis and non-compliance attributions on therapeutic alliance processes in adult acute psychiatric settings. J Psychiatr Ment Health Nurs.

[9] Sansone RA, Sansone LA (2013). Responses of mental health clinicians to patients with borderline personality disorder. Innov Clin Neurosci.

[10] Deans C, Meocevic E (2006). Attitudes of registered psychiatric nurses towards patients diagnosed with borderline personality disorder. Contemp Nurse.

[11] Commons Treloar AJ (2009). A qualitative investigation of the clinician experience of working with borderline personality disorder. NZ J Psychol.

[12] Krawitz R (2004). Borderline personality disorder: attitudinal change following training. Aust N Z J Psychiatry.

[13] Commons Treloar AJ (2009). Effectiveness of education programs in changing clinicians’ attitudes toward treating borderline personality disorder. Psychiatr Serv.

[14] Shanks C, Pfohl B, Blum N, Black DW (2011). Can negative attitudes toward patients with borderline personality disorder be changed? The effect of attending a STEPPS workshop. J Pers Disord.

[15] Banerjee P, Duggan C, Huband N, Watson N. Brief psycho-education for people with personality disorder – a pilot study. Psychol Psychother. 2006;79:385–94. 10.1348/147608305X57987 16945198

[16] Murray-Swank AB, Dixon L (2006). Family psycho education as an evidence-based practice. CNS Spectr.

[17] Zanarini MC, Frankenburg FR (2008). A preliminary randomized trial of psychoeducation for women with borderline personality disorder. J Pers Disord.

[18] Long C, Fulton B, Dolley O (2015). Using psychoeducation to motivate engagements for women with personality disorder in secure settings. J Psychiatric Intensive Care.

[19] Gunderson JG, Berkowitz C, Ruiz-Sancho A (1997). Families of borderline patients: A psychoeducational approach. Bull Menninger Clin.

[20] Knaak S, Modgill G, Patten SP (2014). Key ingredients of anti-stigma programs for healthcare providers: A data synthesis of evaluative studies. Can J Psychiatry.

[21] Pettigrew TF, Tropp LR, Wagner U, Christ O (2011). Recent advances in intergroup contact theory. Int J Intercult Relations.

[22] Pettigrew TF (1998). Intergroup contact theory. Annu Rev Psychol.

[23] Stuart H (2008). Fighting the stigma caused by mental disorders: past perspectives, present activities, and future directions. World Psychiatry.

[24] Szeto ACH, Dobson KS (2010). Reducing the stigma of mental disorders at work: a review of current workplace anti-stigma intervention programs. Appl Prev Psychol.

[25] Kassam A, Papish A, Modgill G, Patten S (2012). The development and psychometric properties of a new scale to measure mental illness related stigma by health care providers: the Opening Minds Scale for Health Care Providers (OMS-HC). BMC Psychiatry.

[26] Modgill G, Patten SB, Knaak S, Kassam A, Szeto AC (2014). Opening minds stigma scale for health care providers (OMS-HC): examination of psychometric properties and responsiveness. BMC Psychiatry.

[27] Inc SPSS (2009). PAWS Statistics for Windows 18.

[28] Pescosolido BA, Monahan J, Link BG, Stueve A, Kikuzawa S (1999). The public’s view of the competence, dangerousness, and need for legal coercion of persons with mental health problems. Am J Public Heal.

[29] Pescosolido BA, Martin JK, Long JS, Medina TR, Phelan JC, Link BG (2010). “A disease like any other”? A decade of change in public reactions to schizophrenia, depression, and alcohol dependence. Am J Psychiatry.

[30] Patten SB, Remillard A, Phillips L, Modgill G, Szeto AC, Kassam A (2012). Effectiveness of contact-based education for reducing mental illness-related stigma in pharmacy students. BMC Med Educ.

[31] Papish A, Kassam A, Modgill G, Vaz G, Zanussi L, Patten S (2013). Reducing the stigma of mental illness in undergraduate medical education: A randomized controlled trial. BMC Med Educ.

[32] Szeto AH, Luong D, Dobson K (2013). Does labeling matter? An examination of attitudes and perceptions of labels for mental disorders. Soc Psychiatry Psychiatr Epidemiol.

[33] Rossberg JI, Karterud S, Pedersen G, Friis S (2008). Specific personality traits evoke different countertransference reactions: an empirical study. J Nerv Ment Dis.

[34] Schulze B, Janeiro M, Kiss H (2011). Does context matter? Illness-specific variations in experiencing and managing stigma between people with schizophrenia and borderline personality disorder. Psychiatr Prax.

[35] Link B, Phelan J (2001). Conceptualizing stigma. Annu Rev Sociol.

[36] Stuart H, Arboleda-Flórez J (2012). Sartorius N.

